# Long-term outcomes of intravitreal therapy for symptomatic diabetic macular oedema in a real-world setting in Switzerland

**DOI:** 10.1007/s00417-021-05187-z

**Published:** 2021-05-04

**Authors:** Johanna J. Zirpel, Isabel B. Pfister, Christin Gerhardt, Justus G. Garweg

**Affiliations:** 1 Berner Augenklinik Am Lindenhofspital, Bremgartenstrasse 119, CH – 3012 Bern, Switzerland; 2 Swiss Eye Institute, Rotkreuz, Switzerland; 3 University of Bern, Bern, Switzerland; 4 Department of Ophthalmology, Inselspital, Bern, Switzerland

**Keywords:** Diabetic macular oedema (DME), Anti-VEGF, Ranibizumab, Aflibercept, Long-term treatment, Consecutive case series, Real-world studies

## Abstract

**Objective:**

To assess the long-term visual outcomes in eyes with symptomatic diabetic macular oedema (DME) under intravitreal treatment (IVT) in a clinical routine setting.

**Methods:**

Patients with newly diagnosed DME were included in this retrospective study if they had received at least three IVTs and a follow-up period ≥ 2 years. Due to altered treatment patterns since the approval of ranibizumab for DME in 2012, patients were subdivided according to their first IVT before 2013 (group 1) or thereafter (group 2). The primary outcome measure was the evolution of best-corrected visual acuity (BCVA) over time.

**Results:**

Of 217 eyes (191 patients) with DME, 151 eyes (117 patients) fulfilled the inclusion criteria (63 eyes in the first period, 88 in the second period). Mean follow-up time was 7.9 ± 3.1 (group 1) and 4.1 ± 1.4 years (group 2; *p* < 0.001). Visual gains were similar in the first year (group 1: + 5.3 ± 15.5, group 2: + 7.3 ± 12.2 Early Treatment Diabetic Retinopathy Study (ETDRS) letters; *p* = 0.44), but not thereafter (after 2 years in group 1: + 4.4 ± 15.0, group 2: + 8.3 ± 13.0 ETDRS letters; *p* = 0.038). During the first year, group 1 patients received less clinical examinations (group 1: 6.6 ± 3.3, group 2: 7.5 ± 2.1; *p* = 0.007) and less injections (group 1: 3.6 ± 2.7, group 2: 6.1 ± 2.7; *p* < 0.001).

**Conclusion:**

A greater visual gain, in response to more intensive treatment during the first year, was maintained for at least 5 years in group 2 subjects. Our data confirm that in a real-world setting, early intensive treatment results in satisfying long-term visual outcomes.

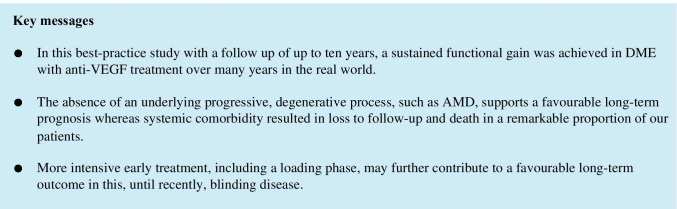



## Introduction

Diabetic macular oedema (DME) is the most common cause of severe vision loss in people aged 20–70 years and the second most common cause of visual impairment [1, 2]. Until the early twenty-first century, 11% of diabetic patients in Western Europe presented with eye disease and 0.3% eventually became blind [1, 3]. Since then, the potential of anti-VEGF drugs to prevent major vision loss and restore reading and driving vision in the majority of patients with diabetic maculopathy has been demonstrated, if the drugs are administered promptly [4].

Diabetic macular oedema (DME) is the most common cause of severe vision loss in people aged 20–70 years and the second most common cause of visual impairment [1, 2]. Until the early twenty-first century, 11% of diabetic patients in Western Europe presented with eye disease and 0.3% eventually became blind [1, 3]. Since then, the potential of anti-VEGF drugs to prevent major vision loss and restore reading and driving vision in the majority of patients with diabetic maculopathy has been demonstrated, if the drugs are administered promptly [4].

Recent evidence indicates that the outcomes from real-world studies are significantly poorer than those from controlled, prospective trials [15–19]. This may be linked to the absence of patient selection criteria, for example, pre-existing structural damage to the macula, and adherence to treatment in real-world settings [16, 20]. The majority of real-world studies in DME provide follow-up of only 2–3 years and a potential bias associated with availability of anti-VEGF drugs to patients. In Switzerland, the intensity of treatment for DME is not limited by the health insurance system. We therefore aimed to assess the long-term real-world treatment outcomes in patients with DME since the introduction of anti-VEGF therapy at our institution, making use of all available therapies and therapy combinations applied according to a best clinical practice pattern.

## Patients and methods

In this single-centre, retrospective, consecutive case series, we included treatment-naïve patients with type 1 and type 2 diabetes mellitus who had received IVT for symptomatic, centre-involving DME at the Berner Augenklinik am Lindenhofspital in Bern, Switzerland.

Inclusion criteria were as follows: [1–4] a minimum follow-up of 2 years at our institution after the first intravitreal therapy.

The presence of any other macular disease (e.g. age-related macular degeneration (AMD) or retinal vascular disease of other origin that could interfere with the clinical outcome, proliferative diabetic retinopathy or absence of centre-involving maculopathy without vision loss), treatment of DME with intravitreal corticosteroid therapy only or as a first-line therapy or non-compliance with the suggested treatment resulted in exclusion from the analysis.

Patients were grouped depending on the date of first injection (between 2007 and 2012 for group 1 and between 2013 and 2017 for group 2). This grouping was based on a change in treatment approach at our institution by the end of 2012 from a minimally needed or pro re nata (PRN) therapy to more sustained therapy aimed at stabilising the early visual gains. This change was possibly based on the cost coverage of intravitreal therapy in DME by health insurance companies since late 2012. Group 2 resembles the current practice pattern following a treat and extend protocol, but not with a fix loading phase of 5 monthly injections, whereas the difference in outcomes compared to group 1 would reflect the impact of medical learning and health cost coverage.

According to the institutional protocol, monthly anti-VEGF injections (either ranibizumab or aflibercept, but since 2012 due to cost coverage problems not bevacizumab) were performed until a significant reduction of intraretinal fluid was achieved, thereafter on an as needed basis (PRN strategy) under monthly to two-monthly visits. Retreatment was guided by anatomic criteria using SD-OCT aiming at functional and morphological stability until 2015. Since then, treatment strategy followed a treat and extend protocol with extension of visits and treatment intervals by 2 weeks until maximally 14 weeks if no new or recurrent fluid was present in OCT. A treatment interruption was offered in the absence of intraretinal fluid over more than 6 months. During therapy pausing, eyes were followed up minimally every 8 weeks according to patient availability.

The study was approved by the Institutional Ethics Committee, University of Bern (reference KEK 2017–00,143) and was carried out in accordance with the Declaration of Helsinki for medical research involving human subjects. Informed consent was obtained from all participants for use of their coded data.

Loss to follow-up was defined as non-compliance with the proposed treatment and/or missing clinic visits for more than 6 months for any reason (treatment futile, patient wishes, change of treating physician, illness or death). The end of follow-up and end of active treatment were defined as the date of the last clinic visit. The follow-up interval was the time from first injection to the last clinic visit.

### Data acquisition

Patient data were retrieved from their electronic records and optical coherence tomography (OCT) database entries linked to patient corresponding visits. Data collected included Snellen’s BCVA, which was converted to the corresponding ETDRS letter scores (study with 85 letters representing a Snellen BCVA of 1.0 and 4 letters representing a BCVA of 0.02), intraocular pressure (IOP) and functionally relevant anatomical findings.

Central foveal thickness (CFT) was measured using a horizontal line algorithm with a length of 6 mm (Spectralis™, Heidelberg Instruments, Heidelberg, Germany). CFT was measured manually from the inner retinal surface to Bruch’s membrane on a micrometre scale.

Data were collected from the time of diagnosis until the last clinical examination before the study data lock on 20 July 2019. This resulted in a maximum of 13 junctures for measurement, including at the time of diagnosis, 1, 6 and 12 months after diagnosis, and annually thereafter, up to a maximum of 10 years. Comparisons between the groups were limited to 6 years of follow-up. Due to the retrospective nature of this study, we accepted a time window of 15% for the coincidence of the clinic visits with the study time junctures. The multiple imputation technique was applied for missing data only in patients still under follow-up at the time of study end [21].

This study was approved by the Institutional Ethics Committee, University of Bern (reference number: 330/14). All patients gave informed consent for the use of their data prior to inclusion in the study, strictly adhering to the tenets of the Declaration of Helsinki.

### Statistical analyses

A series of non-parametric tests were conducted since data were not normally distributed. To compare the temporal profiles of BCVA and central retinal thickness (CRT) between the groups, the Mann–Whitney *U* test was performed. To compare nominal scaled data, the *X*^2^ test was used. Multiple imputation was performed to estimate missing values in group 2 (since many of the patients had not yet reached 5 or 6 years of follow-up). Only the missing data from patients in group 2 still under therapy were imputed. Multiple imputation, as proposed by Rubin in 1978 [22], is a Monte Carlo method of handling data missing at random. It was assumed that any systematic difference between the missing and observed values could be explained by differences in observed data. Multiple imputations are simulated draws from the posterior distribution of missing data. Furthermore, a complete case analysis was also conducted and compared to the findings of the imputed data. The complete case analysis was based only on those individuals without missing data. As Carpenter and Kenward proposed in 2008, it is helpful to present this analysis alongside the analysis performed on the partially observed data to evaluate how the conclusions differ [23]. All statistical analyses were performed using the SPSS software package V.23 (SPSS, Inc., Chicago, IL, USA). A *p* value < 0.05 was considered statistically significant. Unless otherwise stated, the data are presented as mean values and standard deviations (SD).

## Results

Of 217 treatment-naïve eyes (191patients) that received anti-VEGF therapy for centre-involving DME, 66 eyes were excluded from the study (42 treated with corticosteroids as a first-line therapy, 17 had less than 1 year of follow-up, 6 underwent less than 3 injections and one patient was also affected by AMD). Therefore, 151 eyes were eligible for the present study.

Of these included 151 eyes, 63 (41.7%) received their first injection between 2007 and 2012 (group 1) and 88 (58.3%) were treated thereafter (group 2). The groups were similar in terms of mean age at inclusion (group 1: 64.3 ± 13.4 [29.9–90.7] years, group 2: 63.7 ± 13.7 [26.1–92.5] years; *p* = 0.91) and gender (42.9% versus 31.8% female respectively, *X*^2^ test *p* = 0.18). The mean follow-up time differed as per protocol (group 1: 7.9 ± 3.1 [1.9–13.4] years, group 2: 4.1 ± 1.4 [1.8–7.2] years; *p* = 1.38e-13). In group 1, 82.5% were still under therapy after 5 years compared to 70.5% in group 2 (*p* = 0.12). Reasons for loss to follow-up are displayed in Table 1.

**Table 1:** Reasons for loss to follow-up over a follow-up time of 5 years

	First injection	*n* (% of all eyes)	Back to private ophthalmologist	Deceased	Severe systemic disease	Change of home address	Unknown reason
Group 1	2007–2012	11 (17.5%)	45.4%	18.2%	18.2%	-	18.2%
Group 2	2013–2017	26 (29.5%)	69.3%	11.5%	7.7%	7.7%	3.8%

**Fig. 1:**
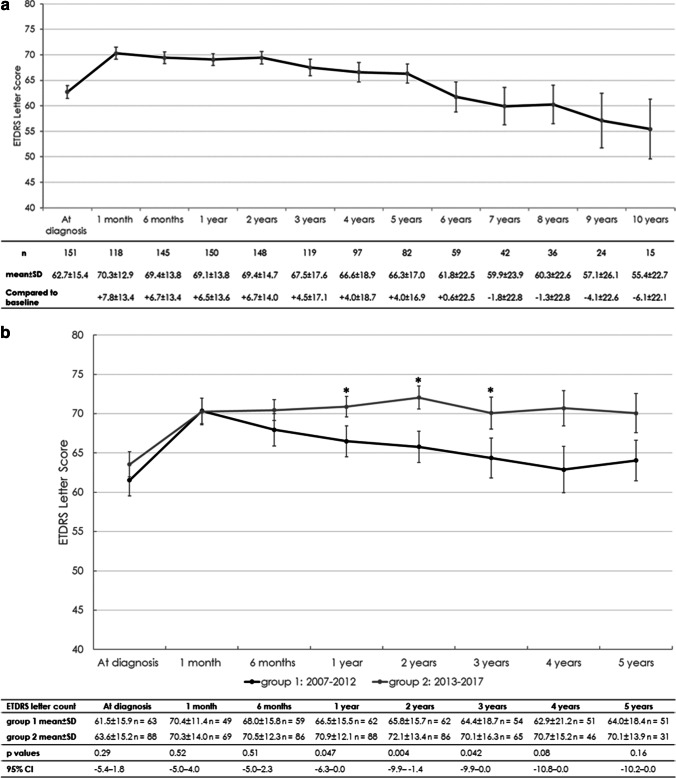
**a** Evolution of best-corrected visual acuity (BCVA) over time (in ETDRS letters with 85 letters representing a BCVA of 1.0; mean ± standard error (SE)) in the full cohort. The distinctive loss in BCVA after 7 years of follow-up is likely related to an inherent selection bias. All patients not further systematically requiring treatment were referred back to their private ophthalmologists until DME recurrence. **b** Evolution of best-corrected visual acuity (BCVA) over time (in ETDRS letters; mean ± standard error (SE)) in two groups representing the periods from 2007 to 2012 and from 2013 to 2017

The pooled group achieved a gain of + 7.8 ± 13.4 (− 30 to + 61) letters after 1 month, which was widely maintained by the end of the second year. After 2 years of follow-up, the letter gain was + 6.7 ± 14.0 (− 30 to + 70) ETDRS letters, which decreased to + 4.5 ± 17.1 (− 49 to + 73) ETDRS letters after 3 years and remained stable until 5 years of follow-up (+ 4.0 ± 16.9 [− 38 to + 74]) (Fig. 1a). Baseline mean BCVA was similar between the groups (*p* = 0.29) (Fig. 1b). The visual gain 1 month after the first injection was similar between the groups (group 1: + 8.4 ± 15.1 [− 23 to + 60] letters, group 2: + 7.4 ± 12.3 [− 30 to + 61] letters; *p* = 0.99). This gain, however, was not maintained in group 1. The letter gain compared to baseline declined to + 5.3 ± 15.5 (− 30 to + 68) letters by the end of the first year, further to + 4.4 ± 15.0 (− 30 to + 70) letters by the second year and finally to + 2.4 ± 18.0 (− 38 to + 75) by year 5. On the other hand, the visual gain in group 2 increased to + 7.3 ± 12.2 (− 15 to + 63) letters by the end of the first year of therapy (*p* = 0.44) and to + 8.3 ± 13.0 (− 30 to + 53) letters by the end of the second year. In group 2, vision remained stable thereafter until 5 years of follow-up (+ 6.6 ± 14.8 [− 23 to + 60] letters) (Fig. 1b). After 1 year, 12.7% of eyes experienced a ≥ five-letter loss. Of the 82 eyes still under our follow-up after 5 years, 20 (24.4%) had lost ≥ five letters. In terms of BCVA, the groups differed over up to 3 years, but the difference in letter gains was significant only at year 2 (*p* = 0.038) (Fig. 1b). The differences among the two groups regarding visual acuity were confirmed for years 2 and 3, whether the missing data were imputed or not. Compared to group 2, more patients who started their therapy between 2007 and 2012 experienced a vision loss of ≥ five letters (year 1: 19.4% versus 8.0% [*p* = 0.048], year 4: 32% versus 8.7% [*p* = 0.006]).

Stratification of visual acuity improvement was performed according to baseline visual acuity (baseline BCVA ≤ 0.5, > 0.5; 70 ETDRS letters) and number of injections in the first year. Patients with a baseline BCVA up to 70 letters demonstrated a stronger visual improvement at all time points (visual gain + 6.2 to + 9.1 letters [baseline visual acuity ≤ 0.5] versus − 2.3 to + 1.7 letters [baseline BCVA > 0.5]) for each year (*p* < 0.001; Table 2). When excluding eyes that had received intravitreal corticosteroids and stratifying the remaining ones by number of injections in the first year (1–4 injections compared to five or more injections), eyes with five or more injections in the first year had only a slightly more pronounced improvement in visual acuity (reaching significance only after 2 years).

**Table 2:** Change in visual acuity (VA) over time, stratified by baseline VA

	*n* (baseline)	1 year	2 years	3 years	4 years	5 years
BCVA ≤ 0.5	104	+ 9.1	+ 8.9	+ 6.2	+ 6.4	+ 6.6
BCVA > 0.5	47	+ 0.60	+ 1.7	+ 0.9	-1.5	-2.3
Comparison (Mann–Whitney U test)		*p* = 2.92e-06	*p* = 3e-04	*p* = 0.024	*p* = 0.015	*p* = 0.02
95% confidence interval		4.85–10.21	3.04–9.86	0.34–9.84	1.63–12.15	1.29–13.11

*BCVA* best-corrected visual acuity

**Fig. 2:**
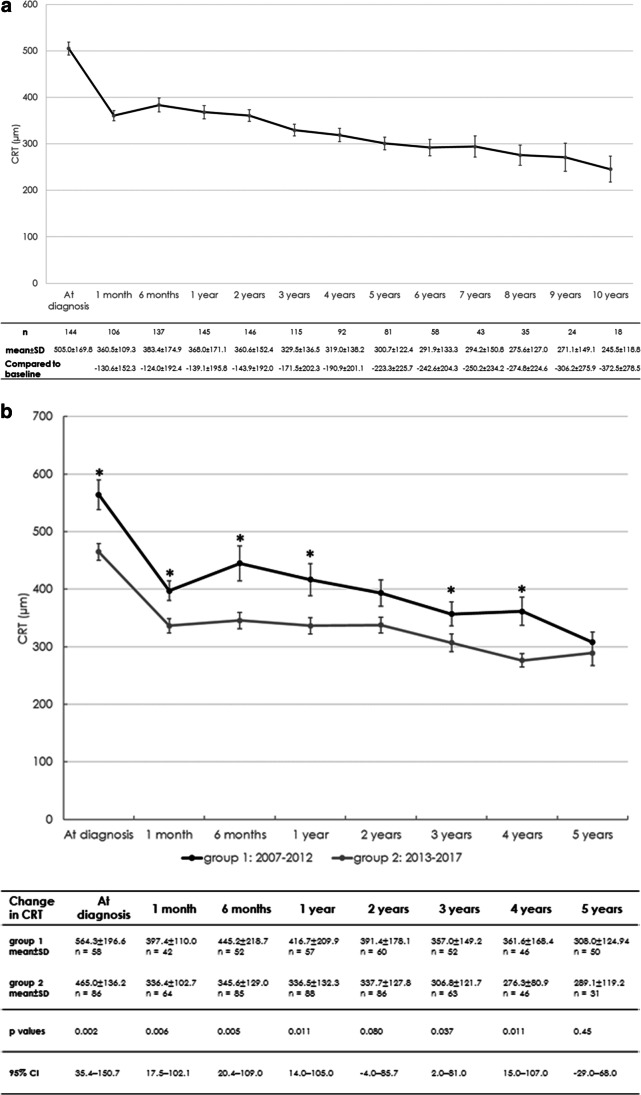
**a** Change in central retinal thickness (CRT) in the full cohort over time (mean ± standard error (SE)). **b** Change in central retinal thickness (CRT) over time in the different time periods (mean ± standard error (SE)): the more pronounced macular oedema at diagnosis in group 1 provides evidence for a late referral early after introduction of this therapy

In contrast to visual acuity, CRT responded well to treatment and stabilised at around 300 (120–889) µm in the overall group between 3 and 10 years of follow-up (Fig. 2a). CRT at baseline was less in group 2 eyes (group 1: 564.6 ± 196.6 [241.7–1045.8] µm, group 2: 465.0 ± 136.2 [224–799] µm; *p* = 0.002) and, most probably related to earlier referral, remained less in group 2 over the following years (Fig. 2b). The imputation of missing data confirmed the differences between the two groups. In line with the PRN protocol in use, a dry macula (absence of any intraretinal fluid) was registered in 18.6% and 18.2% of eyes in group 1 and 2, respectively, after 12 months (*p* = 1.0), as displayed in Table 3.

**Table 3:** Percentage of eyes with residual intraretinal fluid within the macula, including only patients still under treatment. The *p* values for group comparisons are annotated after the values of group 2

	Group 1: 2007–2012				Group 2: 2013–2017			
		Residual intraretinal fluid (%)	Number of visits (mean ± SD)	Number of injections (mean ± SD)		Residual intraretinal fluid (%)	Number of visits (mean ± SD)	Number of injections (mean ± SD)
1 year	*n* = 63	81.4	6.6 ± 3.3	3.6 ± 2.7	*n* = 88	81.8 (*p* = 1.0)	7.5 ± 2.1 (***p = 0.007***)	6.1 ± 2.7 (***p = 2.11e-08***)
2 years	*n* = 63	74.2	6.0 ± 3.3	1.6 ± 1.7	*n* = 88	87.2 (*p* = 0.053)	5.4 ± 2.3 (*p* = 0.13)	3.4 ± 2.7 (***p = 3.94e-05***)
3 years	*n* = 57	75.9	5.2 ± 3.0	1.8 ± 1.7	*n* = 65	75.0 (*p* = 1.0)	4.8 ± 2.1 (*p* = 0.77)	2.9 ± 2.8 (*p* = 0.09)
4 years	*n* = 57	70.8	5.5 ± 3.3	1.4 ± 1.9	*n* = 52	76.1 (*p* = 0.64)	4.5 ± 1.9 (*p* = 0.30)	2.6 ± 2.7 (***p = 0.039***)
5 years	*n* = 53	68.0	5.1 ± 3.4	1.6 ± 1.8	*n* = 31	77.4 (*p* = 0.45)	4.8 ± 2.1 (*p* = 0.86)	3.6 ± 3.7 (***p = 0.028***)

*p* values for group comparisons are annotated after the values of group 2. Significant group comparisons are highlighted in bold

*SD* standard deviation

All group 1 eyes received ranibizumab as first-line therapy. From 2014, treatment in 11 of 63 eyes was switched to aflibercept, while ranibizumab treatment was continued in 52 eyes. Treatment in group 2 eyes commenced with ranibizumab (*n* = 45) or aflibercept (*n* = 41). In the pooled group, 47 patients (31.1%) also received up to three bevacizumab injections in this series (group 1: 39 patients, group 2: eight patients) before reimbursement of ranibizumab for the treatment of DME in Switzerland was instituted.

Group 1 patients received on average 3.6 ± 2.7 [1–13] injections in the first year, while group 2 patients received 6.1 ± 2.7 [1–12] injections during the same time period (*p* = 2.11e-08). This goes along with a higher number of examinations in group 2 in the first year (group 1: 6.6 ± 3.3 [1–17] examinations, group 2: 7.5 ± 2.1 [4–12] examinations; *p* = 0.007). The number of examinations and injections decreased during follow-up (Table 3). Differences between the groups may partially be explained by the use of corticosteroids, namely in the period of time before 2012. In group 1, more than half of the patients had at least one treatment with steroids (group 1: 40 (63.5%), group 2: 27 (30.7%); *p* < 0.0001; Table 4). In the full cohort, patients who had at least one steroid injection during the first year had a lower baseline visual acuity, whereas there was no difference in visual gain after 1 year.

**Table 4:** Number of patients receiving intravitreal steroid injections in the first year and change in visual acuity (VA)

	*n*	Baseline VA mean ± SD	VA change after 1 year mean change ± SD
Steroids in the 1st year	48	55.9 ± 15.2	+ 7.5 ± 16.3
No steroids in the 1st year	103	65.1 ± 14.6	+ 6.0 ± 12.3
Mann–Whitney *U* test		*p* = 1.66e-06	*p* = 0.34
95% confidence interval		4.9 to 15.1	− 6.3 to 3.2

## Discussion

Since the first days of anti-VEGF use in clinical routine, patients enjoyed a relevant visual gain. This explains the high patient adherence over time in clinical practice, which adds to the strengths of our study. A relevant time effect between the two treatment periods may reflect an increasing confidence with the use of intravitreal therapy in DME and a change in the reimbursement pattern with full cost coverage since late 2012 in Switzerland. Best practice has changed in this period. The use of corticosteroids, which are cost-effective but prone to side effects, has declined compared to a more intensive use of on-label anti-VEGF drugs. Increasing knowledge of treatment options resulted in earlier referral of patients, as indicated by a lower retinal thickness at treatment initiation in the second period. In contrast, the impact of an intensified early treatment during the first year on visual gains [8, 24, 25] and improved diabetic retinopathy severity scores (DRSS) with anti-VEGF drugs had not been demonstrated until late 2013 [13, 14] and thus, may have had less impact. A decline in treatment demand over time in our patients is in accordance with published evidence [9, 12]. In a prospective clinical study setting, patients not able or willing to attend regular monthly visits during the first year of treatment would not likely be included in the study. In a real-world setting, young patients in their working lives will seek assistance in order to maintain their daily working activities, but cannot afford frequent visits due to the risk to their employment. These points may at least partially explain the less favourable outcomes in this real-world setting compared to clinical trials. In comparison, the lower adherence to treatment in the second period in our institution reflects an increasing trend to seek anti-VEGF treatment and follow-up by the patients’ family ophthalmologists, rather than a treatment interruption (Table 1) [26]. A more restricted use of triamcinolone and focal and pan-retinal laser photocoagulation and their inherent side effects on visual performance may have added to reduce the treatment burden and patient fears, and thus, have added to the treatment adherence in real-world settings. In addition, patients may also have gathered confidence that their disease, until recently blinding [1, 2, 27, 28], was well treatable in a vast majority of instances with this therapy [8].

Early in the era of anti-VEGF treatment, we aimed to prevent blinding and to preserve some vision in DME in the long term and applied all available therapeutic options to achieve this. Corticosteroids were widely used based on the low cost of off-label triamcinolone compared to ranibizumab (registered for several years for AMD), but neither were approved nor reimbursed for DME. Consistently, the visual gain achieved after the first injection and by end of the first year was not maintained in group 1 compared to group 2. Moreover, 28% of group 1 patients experienced a significant vision loss (> 15 letters) during the first year, which was not linked to cataract development, compared to 8% in eyes treated since 2013. The functional evolution was partially paralleled by morphological parameters, evidenced by a reduced CRT in the second period than the first (Fig. 2b). A patient retention rate of 70–80% over 5 years in both groups adds to the 1-year findings of two prospective studies on high patient satisfaction [29, 30].

One prospective, randomised, interventional, multicentre clinical trial showed that the visual improvement achieved over 6 months in DME patients treated with ranibizumab, with or without additional laser therapy, could be stabilised for up to 2 years of follow-up [25]. Those who were treated with ranibizumab monotherapy gained + 7.2 letters within the first 6 months, compared to 6.7 letters in our study with a significantly lower number of injections. This letter gain was maintained in the prospective setting, as in our real-world study, over 2 years and remained stable at between + 6.6 and 7.7 letters [25]. The remarkable difference in the number of injections between the prospective study with monthly injections, compared to a total of 7.8 injections over 2 years in our patients, is comparable to the RESTORE study findings [10]. The difference in the number of injections and visual gain between our two groups of eyes indicates that a follow-up of 2 years must not be predictive of long-term functional stability. Patients in the second period treated more intensely in the first year maintained their visual gain for up to 5 or more years, whereas undertreatment may explain the progressive vision loss in the first group (Fig. 1b, Table 3). This is well in line with treatment experience in other retinovascular disorders, i.e. retinal vein occlusion [31, 32].

Our real-life study confirms that visual acuity can be maintained for at least 5 years without any central laser treatment if patients are systematically treated with anti-VEGF agents, as long as retinal fluid is present. The assumption that laser therapy might reduce the number of anti-VEGF injections and DME severity [28] has not been confirmed [10, 33–35].

Our data show a remarkable decline in the number of patients still requiring active treatment over a 5-year period, which was also observed in the extension of the RISE and RIDE studies [36]. This suggests that anti-VEGF treatment for DME induces vascular wall stabilisation, which benefits the eye far beyond the direct treatment effect, although over a probable limited period of time. This differentiates DME from the chronically progressive and destructive process of neovascular AMD [18, 19].

One limitation of our study is the low number of eyes that were still under our treatment after 7 years. This is the result of our institutional treatment standard proposing a referral of patients back to their family ophthalmologists after reaching visual stability. Most patients lost to follow-up had been sent back to their family ophthalmologists due to visual stability (45.5% in group 1, 69.2% in group 2; *p* = 0.12). A portion of 45–69% of patients achieving functional stability, on the other hand, underlines the importance of anti-VEGF therapy on long-term stabilisation of retinal function. Since we have no information about the fate of eyes after referral back to family ophthalmologists, our results are probably not representative beyond this time point. Last observation carried forward (LOCF) is an imputation technique used in prospective clinical studies to carry the last assessed value forward to visits where a value is missing. Owing to the retrospective character and less rigid selection criteria, we preferred not to apply this technique to patients in this study whose treatment was suspended. Consequently, a selection bias towards the “more complex” patients with a persisting treatment demand due to chronic recurrent or refractory macular oedema and incomplete response to treatment may explain the vision loss after 6–7 years of treatment. Hence, this does not reflect our impression of the long-term outcome of anti-VEGF treatment in DME.

Long-term stabilisation appears feasible in the majority of DME patients in the real-world setting obviously to a higher degree than in exudative AMD, in which one-third of patients maintain a driving vision over 10 years [37]. This is important since patients with DME are significantly younger than those with AMD and frequently both eyes are affected. Moreover, in contrast to neovascular AMD, continuous treatment of DME eyes appears not to be necessary in a large portion of eyes.

## Conclusion

A sustained functional gain may be achieved in DME with anti-VEGF treatment over many years in the real world. The absence of an underlying progressive, degenerative process, such as AMD, supports a favourable long-term prognosis, whereas systemic comorbidity resulted in loss to follow-up and death in a remarkable proportion of our patients. More intensive early treatment, including a loading phase, may further contribute to a favourable long-term outcome in this, until recently, blinding disease.
